# Epithelial-mesenchymal transition-associated microRNA/mRNA signature is linked to metastasis and prognosis in clear-cell renal cell carcinoma

**DOI:** 10.1038/srep31852

**Published:** 2016-08-23

**Authors:** Hana Mlcochova, Tana Machackova, Anja Rabien, Lenka Radova, Pavel Fabian, Robert Iliev, Katerina Slaba, Alexandr Poprach, Ergin Kilic, Michal Stanik, Martina Redova-Lojova, Marek Svoboda, Jan Dolezel, Rostislav Vyzula, Klaus Jung, Ondrej Slaby

**Affiliations:** 1 Masaryk University, Central European Institute of Technology (CEITEC), Kamenice 5, 625 00, Brno, Czech Republic; 2 Masaryk Memorial Cancer Institute, Department of Comprehensive Cancer Care, Zluty kopec 7, 656 53, Brno, Czech Republic; 3 University Hospital Charite, Humboldt University, Department of Urology, Schumannstrasse 20/21, D-10117 Berlin, Germany; 4 Berlin Institute for Urologic Research, Robert-Koch Platz 7, 10115 Berlin, Germany; 5 Masaryk Memorial Cancer Institute, Department of Diagnostic and Experimental Pathology, Zluty kopec 7, 656 53, Brno, Czech Republic; 6 University Hospital Charite, Humboldt University, Institute of Pathology, Schumannstrasse 20/21, D-10117 Berlin, Germany; 7 Masaryk Memorial Cancer Institute, Department of Urologic Oncology, Zluty kopec 7, 656 53, Brno, Czech Republic

## Abstract

Clear-cell renal cell carcinomas (ccRCCs) are genetically heterogeneous tumors presenting diverse clinical courses. Epithelial-mesenchymal transition (EMT) is a crucial process involved in initiation of metastatic cascade. The aim of our study was to identify an integrated miRNA/mRNA signature associated with metastasis and prognosis in ccRCC through targeted approach based on analysis of miRNAs/mRNAs associated with EMT. A cohort of 230 ccRCC was included in our study and further divided into discovery, training and validation cohorts. EMT markers were evaluated in ccRCC tumor samples, which were grouped accordingly to EMT status. By use of large-scale miRNA/mRNA expression profiling, we identified miRNA/mRNA with significantly different expression in EMT-positive tumors and selected 41 miRNAs/mRNAs for training phase of the study to evaluate their diagnostic and prognostic potential. Fifteen miRNAs/mRNAs were analyzed in the validation phase, where all evaluated miRNA/mRNA candidates were confirmed to be significantly deregulated in tumor tissue. Some of them significantly differed in metastatic tumors, correlated with clinical stage, with Fuhrman grade and with overall survival. Further, we established an EMT-based stage-independent prognostic scoring system enabling identification of ccRCC patients at high-risk of cancer-related death. Finally, we confirmed involvement of miR-429 in EMT regulation in RCC cells *in vitro*.

Clear-cell renal cell carcinoma (ccRCC) is the most common type of kidney cancer and accounts for 60% to 70% of all renal tumors. Despite advances in diagnosis, especially for widespread use of abdominal imaging, and the fact that more than 50% of RCC is detected incidentally, 20–30% of all patients are diagnosed with metastatic disease; another 20% of patients undergoing curative nephrectomy will relapse and develop metastatic RCC with poor prognosis during follow-up^1^,^2^,^3^. Currently, the Czech Republic has the highest incidence and mortality of RCC worldwide with incidence and mortality rates of 15.2 and 4.51 per 100,000^4^.

RCC is characterized by its diverse clinical course. Some RCCs are aggressive and resistant to current therapies, while others are slow-growing^2^,^3^. Although a number of traditional clinicopathologic parameters (e.g. TNM stage, Fuhrman grading, performance status) are used in current clinical practice, the ability to predict the outcome after surgical or systemic therapy is still limited, underscoring the need for new approaches or novel prognostic markers to the management of RCC^3^,^5^. We expect that a combination of specific molecular RCC biomarkers with conventional clinicopathologic characteristics will allow better prediction of prognosis.

Epithelial-mesenchymal transition (EMT) is one of the key processes discussed in regards to progression and metastasis of a wide range of cancers, including RCC^6^. During EMT, polarized epithelial cells undergo multiple biochemical and morphological changes altering their phenotype from epithelial to mesenchymal^7^. Loss or down-regulation of epithelial markers (e.g., E-cadherin, cytokeratins) and up-regulation of mesenchymal markers (e.g., N-cadherin, S100A4, vimentin) are presented as hallmarks of EMT. These modulations result in increased motility, invasiveness and resistance to apoptosis of cancer cells^8^. This way, morphologically changed cells could get released from the primary tumor mass, enter the blood/lymph circulation and spread through the body.

Several transcription factors, including Snail, Slug, Twist, ZEB1, and ZEB2, have been identified as inducers of EMT and tumor metastasis^8^. More recently, miR-205 and the miR-200 family (miR-200a, miR-200b, miR-200c, miR-141, and miR-429, which share a consensus seed sequence) have emerged as new epithelial markers and repressors of EMT^9^ and markers of stem cell properties^8^. The miR-200 family members function to promote MET and inhibit induction of EMT, doing so by directly targeting the mRNAs encoding ZEB1 and ZEB2. Conversely, ZEB1 represses the transcription of miR-200 genes by directly binding to their promoter region, thereby forming a double-negative feedback loop^9^. Involvement of miR-200 family in regulation of EMT was recently described also in RCC^10^.

Although the detailed role of EMT in metastatic cascade, especially in RCC, remains poorly understood, it seems that alterations in miRNA regulatory network are critical for induction of EMT. We believe that a combinatorial approach based on evaluation of miRNAs and genes associated with EMT enables efficient identification of a metastatic phenotype in primary tumors and mainly prognosis prediction in RCC patients. Therefore, the aim of our study was to identify an integrated miRNA/mRNA signature associated with metastasis and prognosis in ccRCC through a targeted approach based on analysis of miRNAs/mRNAs associated with EMT.

## Methods

### Patients and tissue samples

**Figure 1:**
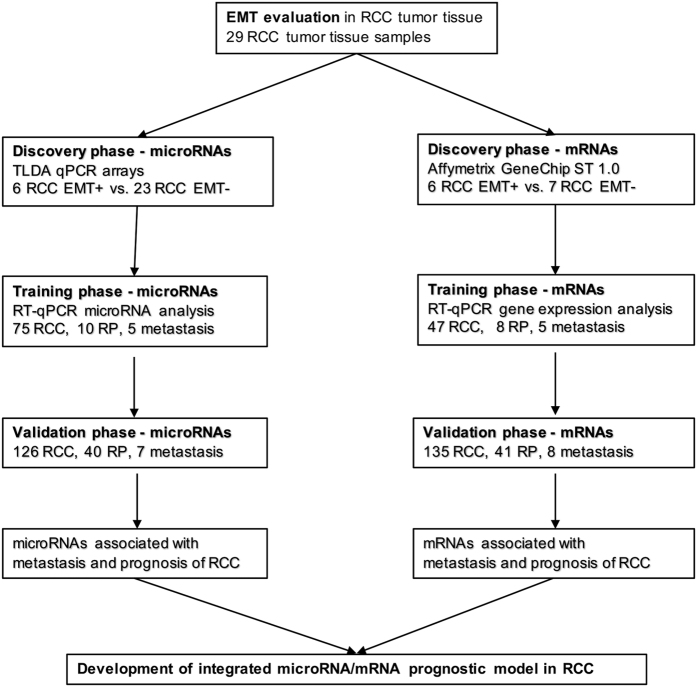
A flow chart of the study design. RP (renal parenchyma), RCC (renal cell carcinoma).

This study was designed as 3-phase monocentric biomarker study (Fig. 1). Frozen and formalin-fixed, paraffin-embedded (FFPE) primary tumor samples, adjacent tissue and metastasis were obtained from patients diagnosed with ccRCC and surgically treated at the Masaryk Memorial Cancer Institute (Brno, Czech Republic) between 2008 and 2013. For EMT evaluation, 29 patients were enrolled, and their FFPE tumor tissue was used for immunofluorescence staining. Another cohort of 201 ccRCC cases was included, which was further divided into the discovery, training and validation cohorts to identify and validate EMT-associated miRNAs/mRNAs and to develop of an integrated miRNA/mRNA prognostic model. All samples were frozen immediately after surgery and stored in liquid nitrogen until RNA extraction. Clinical characteristics of ccRCC patients are summarized in Table 1. All subjects enrolled in the study were of the same ethnicity (European descent), and ccRCC patients did not receive any neo-adjuvant treatment before surgery. Written informed consent was obtained from all patients, and the study was approved by the local Ethics Board at the Masaryk Memorial Cancer Institute. All experiments were performed in accordance with relevant guidelines and regulations.

### Immunofluorescence staining of EMT markers

Immunofluorescence analysis of FFPE tumor tissues was performed using standard staining procedure. In brief, FFPE slides were incubated with primary antibodies at room temperature for 1 hour and washed with 0.025% TBS/Tween and TBS. Slides were incubated with secondary antibodies with Alexa 488-labeled/Alexa 594-labeled for 30 minutes at room temperature and washed with 0.025% TBS/Tween and TBS. After staining, slides were mounted with 3–4 drops of ProLongR Gold Antifade Reagent with DAPI. Antibodies used in the study are listed in Supplementary Table S1. Evaluation was conducted with fluorescence microscope (Leica), and samples were evaluated as described previously. In detail, the staining intensity was graded as follows: 0 (negative), 1 (weak), 2 (moderate) and 3 (strong). The percentage of positively stained cells in the tumor sample was counted in a 0–100% scale, where numbers described percentage positivity of cells. Thus, 0 (0–5%), 1 (6–25%), 2 (26–75%), 3 (76–100%). Ten repeats of each randomly-selected microscope IF fields of tumor sample were counted in 3 independent repeats. The sum of staining intensity + percentage of positively stained cells was calculated as a total number of expression for weak positivity (≤3) and for strong positivity (>3).

### RNA isolation

Native tissue samples were homogenized with tissue homogenizer MagNA Lyser Instrument (Roche Applied Science) in sterile conditions before total RNA isolation. Total RNA was extracted with mirVana™ miRNA Isolation Kit (Ambion) according to manufacturer’s instructions. Total RNA concentration and purity were measured by UV spectrophotometry using Nanodrop ND-1000 (Thermo Scientific). RNA integrity was evaluated using Bioanalyzer 2100 (Agilent) in samples used for whole genome expression profiling (only samples with RIN >8 were used).

### Global miRNA expression profiling

TaqMan Low Density Arrays (TLDA) were used to obtain global miRNAs expression profiles in 29 ccRCC (6 EMT^+^ and 23 EMT^−^) samples. Briefly, 35 ng of total RNA was reverse-transcribed into cDNA using Taq-Man MicroRNA Reverse Transcription Kit and microRNA Megaplex RT set pool A and B version 3.0 (Applied Biosystems). The cDNA product was subsequently loaded into TaqMan Human MicroRNA A and B Cards Set version 3.0 (Applied Biosystems). All reactions were performed according to the standard manufacturers’ protocols on ABI 7900HT Instrument (Applied Biosystems). Samples with tabulated RAW microRNA expression profiles included in this study have been deposited in the ArrayExpress Archive database under accession number E-MTAB-4911, available online (http://www.ebi.ac.uk/arrayexpress/).

### Whole genome expression profiling

Two hundred and fifty ng of total RNA were used for GeneChip^®^ Whole Transcript (WT) Expression Arrays (Affymetrix) according to manufacturer’s protocol. cDNA was hybridized to GeneChip Human Gene 1.0 ST (Affymetrix) at 45 °C for 16 hours. Subsequently, GeneChips were washed and scanned (GeneChip^®^ Scanner 3000 7 G, Affymetrix). The whole-genome expression data, Affymetrix raw data (.cel files), were normalized using the robust multichip average (RMA). Generated CEL files of samples included in this study have been deposited in the ArrayExpress Archive database under accession number E-MTAB-4909, available online (http://www.ebi.ac.uk/arrayexpress/).

### RT-qPCR validation of EMT-associated miRNAs/mRNAs

MiRNAs and genes, which were selected for further evaluation in the training phase and independent validation in validation phases, are listed together with the assays (Applied Biosystems) in Supplementary Tables S2 and S3. Total RNA was reverse-transcribed using Taq-Man MicroRNA Reverse Transcription Kit and High-Capacity Reverse Transcription Kit (both Applied Biosystems) and used for qPCR. qPCR was performed using TaqMan Gene Expression MasterMix (Applied Biosystems) for mRNAs and TaqMan Universal MasterMix II, no UNG (Applied Biosystems) for miRNAs and QuantStudio 12 K Flex Real-Time PCR System according to manufacturer’s recommendations. The quantification cycle (Cq) data were calculated using the default threshold settings (0.2). All miRNA and gene expression values were calculated according to the following formula: 2^−dCq^. Data were normalized to RNU48 in case of miRNA analysis and to PPIA for gene expression analysis. Reference genes (RNU48, PPIA) were selected as an endogenous control through combination of standard geNorm and NormFinder algorithms. All samples were run in duplicates.

### *In vitro* analysis of EMT

Human renal carcinoma cell lines Caki-2 and 786–0 were used in the study. Both cell lines were obtained from the ATCC and maintained in recommended media containing 10% fetal bovine serum, 2 mM glutamax, 100 U/ml penicillin G and 0.1 μg/ml streptomycin. For transfection, 33.3 nM of pre-miR-429 was used (Applied Biosystems). To induce EMT, recombinant human transforming growth factor-β (TGF-β) was used at a concentration of 10 ng/ml (Applied Biosystems). To study EMT process *in vitro*, Caki-2 and 786–0 cells were plated in 24-well plates. One day after seeding, cells were transfected with 33.3 nM of pre-miR-429. EMT was induced using TGF-β a day after transfection as described previously^11^. Morphology of cells was studied 2 days after TGF-β treatment, and cells were harvested with Qiazol for total RNA isolation. CDH1 levels were evaluated by RT-qPCR as described above. Values are presented as means of 3 independent experiments.

### Statistical analysis

Differences between subgroups were tested with Mann–Whitney U-test and Kruskal-Wallis test. Differences between *in vitro* experiments were tested by t-test. Survival analysis were calculated with Kaplan–Meier method using log-rank test. The whole-genome expression data were analyzed using Bioconductor package in R version 3.0.1;LIMMA package was used to identify differentially expressed genes. P-values lower than 0.05 were considered to be statistically significant.

The discovery of integrated miRNA/mRNA prognostic signature was performed using Cox proportional hazard regression. Both cohorts were joined because of the small number of survival events in each cohort separately. Selection of miRNA/mRNA subset with predictive ability was done by 10-fold cross-validation. For each 9/10th of the patients (training set), all 15 miRNAs/mRNAs, which were successfully validated in 3-phase biomarker study, were employed as candidate predictors and used for further stepwise selection into a predictive model within the same fold. The best model for each fold in the training set was chosen by stepwise selection, with forced predictors (age, gender and stage), which repetitively added or dropped miRNAs/mRNAs until minimizing Akaike information criterion. This best model was applied on the validation set in each fold. A final consensus model was derived from 10 respective models obtained within cross-validation and was comprised of miRNAs/mRNAs which were selected in at least 7 of the 10 folds. To obtain upper level for predictive accuracy of the selected consensus proteins, the final model was fit to the full dataset, and the predictive accuracy was quantified using the area under the ROC curve, sensitivity, specificity and accuracy. Moreover, an unbiased estimate of the predictive ability of the selected miRNAs/mRNAs was obtained by the pseudomedian fold of the cross-validation step, which corresponds to the 5th largest area under the curve value out of the 10 folds. An estimate of variability associated with the ROC curve was obtained by plotting the 25th and the 75th quantile of the sensitivities for each value of 1-specificity over ten folds. The predictive ability of the selected model was evaluated using the remaining 1/10th of the patients. The same procedure was repeated 10 times, by systematically selecting different 9/10th and 1/10th of the patients. Finally, a consensus model was formed from the most frequently selected miRNAs/mRNAs in all folds, that is, miRNAs/mRNAs selected in at least 5 of the 10 folds.

All calculations were performed using GraphPad Prism version 6.00 (GraphPad Software, La Jolla, CA) and *R* environment (R Development Core Team). P-values of less than 0.05 were considered statistically significant. Pseudocode of predictive analyses: COX and 5-years overall survival (OS) is provided as supplementary code 1.

## Results

### Evaluation of EMT status in RCC tumor tissue

According to immunofluorescence analysis, EMT status of 29 RCC tumor tissues was determined. Based on CDH1, CK18 and CK19 negativity (low expression) and S100A4 and VIM positivity (high expression), patients were considered to be EMT positive (EMT+, n = 6). Contrary to EMT+, patients positive for epithelial markers and negative or weakly positive for mesenchymal markers represented EMT negative samples (EMT−, n = 23). Results of IF analysis are summarized in Supplementary Table S4. EMT+/EMT− subgroups were used for high-throughput miRNA/mRNA profiling analysis.

### Discovery phase–identification of EMT-associated miRNAs/mRNAs

To obtain miRNAs differentially expressed between EMT+ and EMT− ccRCC samples, global miRNA profiling was employed. Using LIMMA and HCL statistical approach, 26 miRNAs were identified to have significantly different expression levels between the two groups of patients (P < 0.05; Supplementary Table S5). Fifteen miRNAs were selected to be further evaluated in the training phase of the study accordingly to the following criteria: fold-change >2, p < 0.05 and average Cq in one of the tested groups lower than 35. Additionally, miR-200c/miR-141 and miR-30d* were selected for the training phase as other members of the miR-200 family and miR-30 family, respectively, were identified in the discovery phase, and there is literature evidence of their involvement in EMT. Similarly, miR-215 and miR-193b were selected due to their sequence homology and co-regulatory relationship with miR-192 identified in the discovery phase and previously published reports of their involvement in EMT. Total number of 20 miRNAs were analyzed in the training phase of the study: members of miR-200 family (miR-200a, miR-200a*, miR-200b, miR-200b*, miR-200c, miR-429, miR-141), miR-30 family (miR-30a-5p, miR-30a-3p, miR-30b, miR-30c, miR-30d*, miR-30e, miR-30e-3p), miR-130b*, miR-630, miR-17*, miR-215, miR-192* and miR-193b.

Genes differentially expressed between EMT+ and EMT− ccRCC samples were identified by Affymetrix GeneChip arrays for whole genome expression profiling. Based on the requested level of significance, 64 differentially expressed genes (p < 0.005), 27 genes (p < 0.001) or 16 genes (p < 0.0005) were observed. Genes with p < 0.005 are summarized in Supplementary Table S6. Out of this group of genes, 15 EMT-associated genes were selected for further evaluation in the training phase according to the following criteria: fold-change >1.5, p < 0.0015 and average Cq in the one of the tested groups lower than 35. Another 6 common EMT-markers were added (CDH1, CDH12, VIM, S100A4, ZEB1, ZEB2). CDH1, CDH12 VIM and S100A4 were selected for the training phase as those are the most commonly used markers in EMT studies. Transcription factors ZEB1, ZEB2 are involved in the miR-200 family regulatory network. These additional markers were also significantly deregulated in EMT-positive tumors (P < 0.05), but had not been found in the most significant gene group. Total number of 21 genes were analyzed in the training phase of the study: SLC7A5, SLC22A24, SLC25A29, SCD, PAPSS2, C3orf52, FREM1, SEPT14, KMO, ZNF343, CLVS2, CALCR, PCDHB10, SLC17A3, ITGB1BP1, CDH1, CDH12, VIM, S100A4, ZEB1 and ZEB2.

### Training phase

In the training phase of the study, expression levels of 41 EMT-associated miRNAs/mRNAs were evaluated in renal parenchyma and tumor tissue. Results of the miRNAs analysis in the training phase of the study are summarized in Supplementary Table S7. We observed significantly decreased levels of miR-200a, miR-200a*, miR-200b, miR-200b*, miR-200c, miR-429, miR-141, miR-30a-5p, miR-30a-3p, miR-30b, miR-30c, miR-30d*, miR-30e, miR-30e-3p, miR-130b* and increased levels of miR-17* in tumors in comparison to renal parenchyma. Expression of miR-630 was not detectable (average of Cq in both groups >35). Further, 10 miRNAs were significantly associated with Fuhrman grade and 7 miRNAs with TNM stage. More importantly, decreased expression levels of miR-200a, miR-200b, miR-200b*, miR-429, miR-30a-3p, miR-30e and miR-30c were significantly associated with shorter overall survival, indicating a potential prognostic utility of these miRNAs. Based on the differences between studied subgroups (FC, p-value), association with stage, grade and overall survival (at least 2-fold difference in expression and two significant associations with tested factors), we selected 8 miRNAs for independent confirmation in the validation phase of the study: miR-200a, miR-200b, miR-200c, miR-429, miR-30a-5p, miR-30a-3p, miR-30e and miR-30e-3p.

Analysis of gene expression indicated decreased levels of CDH1, CDH12, SLC22A24, SLC25A29, PAPSS2, C3orf52, FREM1 and ITGB1BP1 and increased levels of VIM and SCD in tumors in comparison to renal parenchyma. Expression of SEPT14 was not detectable. Further, 6 genes were significantly linked with Fuhrman grade and 2 genes with TNM stage. More importantly, decreased expression levels of CDH1, SLC25A29, PAPSS2, PCDHB10, C3orf52 and FREM1 were significantly associated with shorter overall survival, indicating potential prognostic utility of these genes. Results of the gene expression analysis in the training phase of the study are summarized in Supplementary Table S8. Based on the differences between studied subgroups (FC, p-value), correlations with stage, grade and overall survival (at least 2-fold difference in expression and two significant associations with tested factors), we selected 7 genes for independent confirmation in the validation phase of the study: CDH1, SLC22A24, SLC25A29, SCD, PAPSS2, C3orf52 and FREM1.

### Validation phase

**Figure 2:**
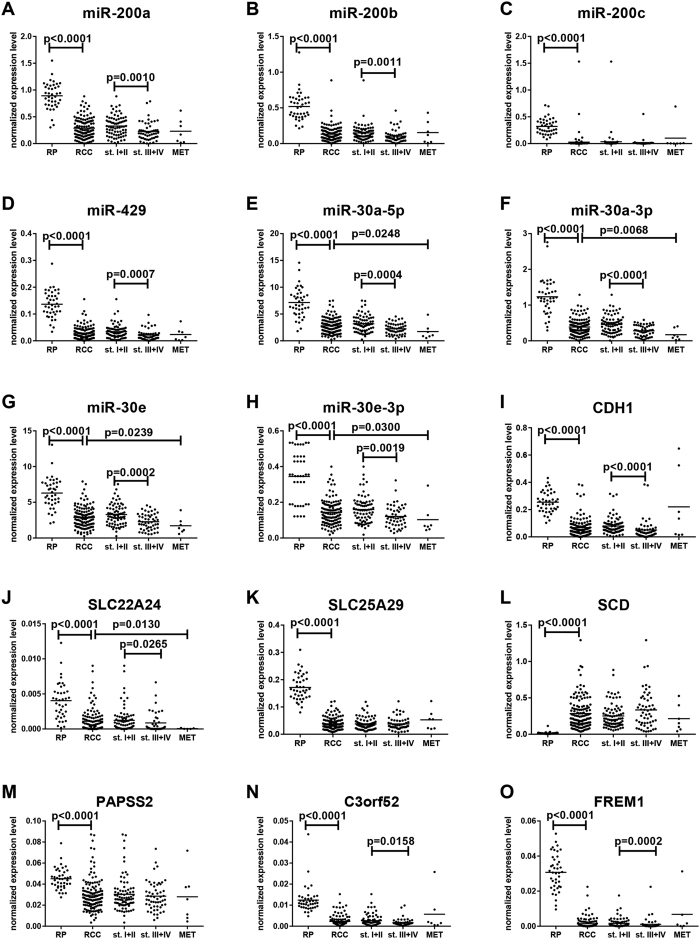
Expression levels of the EMT-associated miRNAs/mRNAs in renal parenchyma, tumors (all TNM stages), non-metastatic tumors (stage I + II), metastatic tumors (stage III + IV) and metastasis (validation phase of the study). All comparisons are between two groups by use of Mann-Whitney test. RP (renal parenchyma), RCC (renal cell carcinoma), st.I + II (TNM stage I + II), st.III + IV (TNM stage III + IV), MET (metastasis).

**Figure 3:**
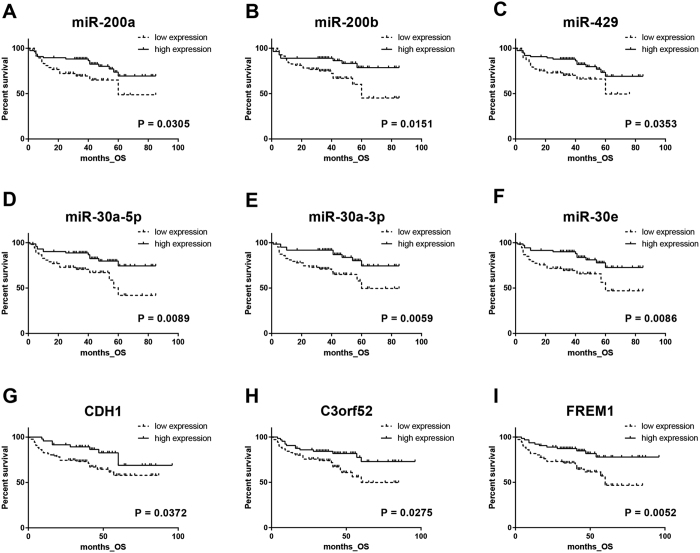
EMT-associated miRNAs/mRNAs in relationship to the overall survival of ccRCC patients in validation phase of the study (cut-off values for prognostic stratification were adapted from training phase of the study).

In the validation phase of the study, 15 EMT-associated miRNAs and mRNAs were studied. We confirmed significantly decreased levels of miR-200a, miR-200b, miR-200c, miR-429, miR-30a-5p, miR-30a-3p, miR-30e, miR-30e-3p, CDH1, exclude, SLC22A24, SLC25A29, PAPSS2, C3orf52 and FREM1 and increased levels of SCD in tumors in comparison to renal parenchyma (all p < 0.0001). Expression levels of miR-200a, miR-200b, miR-429, miR-30a-5p, miR-30a-3p, miR-30e, miR-30e-3p, CDH1, SLC22A24, C3orf52 and FREM1 were significantly decreased in metastatic (TNM stage III with pN+ and IV) compared to non-metastatic tumor (TNM stage I and II) (Fig. 2). Further, 7 miRNAs and 3 genes were significantly associated with Fuhrman grade (Supplementary Figures S1+S2), and 7 miRNAs and 5 genes correlated with TNM stage (Fig. 2). More importantly, when cut-off values for prognostic stratification established at training phase were used, decreased expression levels of CDH1, C3orf52 and FREM1 were significantly associated with shorter overall survival, indicating potential prognostic utility of these mRNAs (Fig. 3). Detailed results of the validation phase of our study are summarized in Table 2A.

### Development of integrated miRNA/mRNA prognostic model

**Figure 4:**
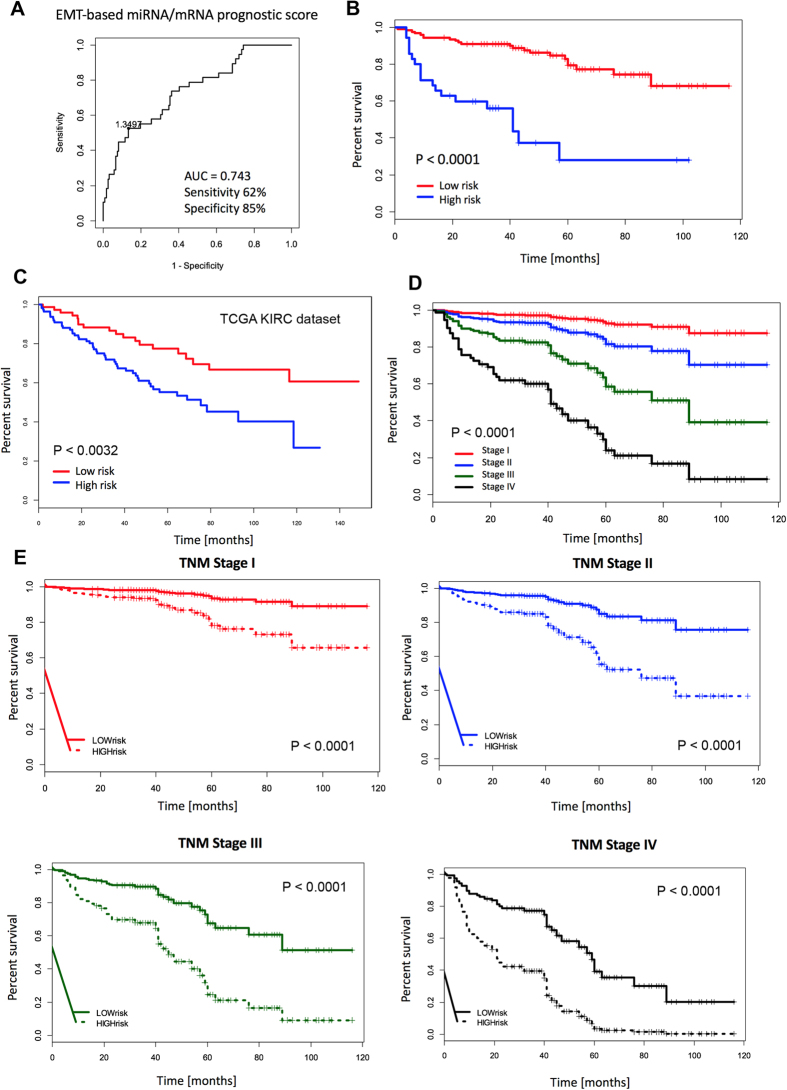
Integrated EMT-based miRNAs/mRNAs prognostic model. The best model for the prediction of 5-year overall survival consisted of 5 miRNAs (miR-200a, miR-200b, miR-200c, miR-429, miR-30a-3p) and 3 genes (C3orf52, CDH1, PAPSS2) (**A,B**). Independent validation of the integrated miRNA/mRNA EMT-signature by use of The Cancer Genome Atlas dataset KIRC (**C**). The predicted survival was assessed for the model without EMT-based miRNA/mRNA signature (**D**) and the model based only on the EMT-based miRNA/mRNA signature (age, gender, stage were fixed values) (**E**).

The association of 15 validated miRNA and mRNA candidates with patient overall survival was examined, and models predicting patients’ outcome were generated. Selection of miRNA/mRNA subset with predictive ability was done by 10-fold cross-validation (Supplementary Table S9). The best signature for prediction of 5-year overall survival consisted of 5 miRNAs (miR-200a, miR-200b, miR-200c, miR-429, miR-30a-3p) and 3 genes (C3orf52, CDH1, PAPSS2) (Fig. 4A,B). Multivariate analyses demonstrated that a high-risk group characterized by risk score above cut-off value (1.3497, corresponds to Youden index), calculated by linear combination of miRNAs/mRNAs expression: 3.75*miR-200a + 2.94*miR-200b–9.84*miR-200c–1.65*miR-30a-3p–53.03*miR-429–196.74*C3orf52–11.22*CDH1–30.25*PAPSS2, is a significantly unfavorable prognostic factor independent of other clinical variables (hazard ratio 3.63; 95% CI 1.69–7.82; P < 0.0009; Table 2B). The unit of particular miRNA/mRNA level is the relative fold-change to endogenous control. The outcome model that included the miRNA/mRNA signature adjusted by the clinical factors explained survival of patients better than the model that included clinical factors alone (likelihood-ratio test, P < 0.0019). To evaluate the contribution of the miRNA signature to the predictive ability, all collected survival data spanning more than 5 years of observations were employed, and the model-based predictions of probability of survival were visualized for each stage independently. Predicted survival was inspected for the model without miRNA/mRNA signature (Fig. 4D), and the model based only on the miRNA/mRNA signature (age, gender, stage were fixed values) (Fig. 4E). A large separation of the predicted survival curves was observed for all stages pointing to an added value of miRNA/mRNA signature for outcome prediction in RCC.

### Validation of the integrated miRNA/mRNA EMT-signature by use of The Cancer Genome Atlas data

We used TCGA-KIRC dataset^12^ of 191 RCC patients for whom overall survival, miRNA and mRNA sequencing expression profiles were available for validation of our EMT-signature. Further, RPKM (the Reads Per Kilobase of exon per million reads Mapped)-normalized mRNAs/miRNAs expression levels were analyzed in a multivariate Cox regression model. The cut-off value (0.8958, corresponds to Youden index for 5-year overall survival) was calculated from risk prediction and the Cox model formula was used to stratify RCC patients from TCGA-KIRC dataset into high-risk (n = 112) and low-risk (n = 79) group. Kaplan-Meier analysis confirmed that overall survival of the high-risk patients was significantly shorter in comparison to low-risk patients (*P* < 0.0032, log-rank test) (Fig. 4C).

### Involvement of miR-429 in EMT regulation *in vitro*

**Figure 5:**
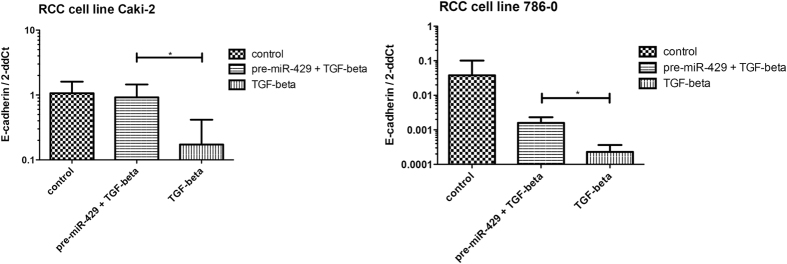
MiR-429 restores TGF-β-induced CDH1 suppression in renal cell carcinoma cell lines. (* means p < 0.05).

As miR-429 is the least studied member of the miR-200 family in EMT and cancer, we decided to evaluate its involvement in regulation of EMT process *in vitro*. To study EMT in renal carcinoma cell lines, cells were treated with TGF-β, which was followed by a strong suppression of CDH1 expression and notable morphological changes of the cells. To evaluate functioning of miR-429 in EMT process induced by TGF-β, RCC cells were transfected with pre-miR-429. One day after transfection, treatment with TGF-β was carried out. After two days, it was observed that cells influenced with TGF-β showed a strong suppression of CDH1 compared to pre-miR-429 transfected cells, where TGF-β effects on CDH1 levels were suppressed or reversed (Caki-2, p = 0.024; 786-O, p = 0.035; Fig. 5).

## Discussion

EMT refers to a morphological transformation of cells that lose their epithelial features and acquire a mesenchymal phenotype. Such transition has been shown to contribute to tumor metastatic potential^7^,^8^. It seems that EMT is significantly associated with prognosis of RCC patients^13^. Among transcription factors, which are critical for induction of EMT, ZEB2 and Snail have been associated with poor overall and progression-free survival of RCC patients^14^,^15^. Also, miRNAs have a significant role in the metastatic cascade, including the EMT process, through regulation of key genes, such as ZEB1, ZEB2, Snail, Twist^16^ etc. However, prognostic role of EMT-associated miRNAs in RCC has not been evaluated until now, and the number of genes studied in connection with EMT is limited. We believe that a combinatorial approach based on evaluation of miRNAs and genes related to EMT enables efficient identification of a metastatic phenotype in primary tumors and mainly prognosis prediction in ccRCC patients with localized disease.

In our study, we evaluated ccRCC tumor tissues by immunofluorescence staining of common EMT markers (CDH1, CK18, CK19, VIM, S100A4) and stratified patients to EMT positive and EMT negative groups. Consequently, in the discovery phase of our study, we identified miRNAs and genes associated with EMT positive tumors. The most significantly deregulated EMT-associated miRNAs were two well-known miRNA families, previously linked to the EMT process: miR-200 and miR-30 family, specifically miR-200a, miR-200b, miR-200c, miR-429,miR-30a-5p, miR-30a-3p, miR-30e and miR-30e-3p. These miRNAs were also successfully validated in the training and validation phase of the study as deregulated in tumor tissue. With the exception of miR-200c and miR-30e-3p, they also correlated with TNM stage, Fuhrman grade and overall survival of ccRCC patients.

Accordingly, the miRNA-200 family (miR-200s: miR-200a/b/c, miR-141, and miR-429), which is an important regulator of tumor invasion and metastasis through its targeting of ZEB1 and ZEB2, has been repeatedly found to be significantly downregulated in RCC^9^,^10^. In RCC, it has been previously shown that the miR-200 family plays an important role in EMT also through modulation of focal adhesion pathways and the ErbB signaling pathway^17^. In addition, *in vitro* experiments showed that stable overexpression of miR-429, another member of miR-200 family, in RCC cell lines, strongly inhibited cell proliferation, colony formation, migration and invasion through direct targeting of BMI1 and E2F3^18^. As a secondary aim of our study, we decided to evaluate the potential of miR-429, the least described member of the miR-200 family, to restore CDH1 levels *in vitro*, and we confirmed the ability of miR-429 to reverse CDH1 suppression induced by TGF-β. Decreased expression of miR-429 was also connected with shorter overall survival overall survival in RCC patients^19^, which is in agreement with our observations.

Increasing evidence suggests that both pre-miRNA strands (leading, passenger) in miRNA biogenesis pathway may lead to production of mature miRNA incorporated in miRISC complex under specific conditions^20^. According to our data, miRNAs maturated from both strands of miR-30a and miR-30e (miR-30a-5p, miR-30a-3p and miR-30e, miR-30e-3p) are present in renal parenchyma and strongly decrease in ccRCC tumor tissue. Supporting our observations, almost the entire miR-30 family (miR-30a,b,c,d) was shown to be downregulated in metastatic RCC tissue^21^. MiR-30a-3p has been proven to have the ability to restore CDH1 expression in hepatocellular carcinoma^22^. Further, it was observed that hypoxia induces EMT in RCC cells through downregulation of miR-30c-5p, which leads to subsequent increase of Slug expression and repression of E-cadherin production^23^.

E-cadherin, which is encoded by CDH1 gene, plays a key role in cellular adhesion and its loss is a fundamental feature of the EMT process^6^,^7^. In our study, CDH1 was one of seven EMT-associated genes (CDH1, SCD, PAPSS2, C3orf52, FREM, SLC22A24, SLC25A29) successfully validated to be deregulated in tumor tissue. Moreover, CDH1 was observed to be progressively downregulated in sequence parenchyma, non-metastatic tumor, metastatic tumor and metastases and also significantly correlated with stage, grade and survival in RCC patients. Moreover, we identified genes involved in cancer metabolic pathways, such as SCD and PAPSS2, in the tumor samples with EMT positive status. SCD (stearoyl-coenzyme A desaturase) correlated with TNM stage and was the only gene within this group with increased expression in ccRCC tumors. SCD is known as the key enzyme in regulation of membrane fluidity and carbohydrate metabolism that fuels tumor growth, increases energetic capacity and invasive and migratory properties of cancer cells^24^. Concordantly with our data, increased expression of SCD in ccRCC tumors was previously described^24^. Targeting metabolic ACSL (acyl-CoA synthetase)/SCD network with chemical inhibitors was suggested to be a promising treatment strategy to inhibit EMT^25^. Another EMT-associated gene observed in our study was PAPSS2 (3′-phosphoadenosine 5′-phosphosulfate synthase 2), known as mesenchymal metabolic gene previously described as a tumor suppressor gene in RCC with significantly downregulated expression in tumor tissue^26^. PAPSS2 loss was observed also in patients with PSA recurrence after radical prostatectomy^27^. C3orf52 (TTMP) was described as a tumor suppressor in pancreatic adenocarcinoma cell line^28^; in our study, it was downregulated in tumor tissue and correlated with stage and overall survival of ccRCC patients. We identified other genes (SLC22A24, SLC25A29 and FREM1) as associated with EMT; they were linked to cancer for the first time. Interestingly, deregulation of these genes in RCC tumor tissue is highly significant and present in almost all cases (Fig. 2). FREM1 is an extracellular matrix protein which is essential for the formation of the epithelial basement membrane during embryonic development^29^ and is widely expressed in the developing embryo in regions of epithelial/mesenchymal interaction and epidermal remodeling^30^. Functioning of SLC22A24 and SLC25A29 remains largely unknown not only in RCC.

To further evaluate the prognostic potential of EMT-related miRNAs/mRNAs, the association of biomarker candidates with patients’ overall survival was examined, and models predicting patients’ outcome were generated. The best EMT-associated miRNA/mRNA signature for the prediction of 5-year overall survival, consisting of 5 candidate miRNAs (miR-200a, miR-200b, miR-200c, miR30a-3p, miR429) and 3 candidate genes (C3orf52, CDH1, PAPSS2), allowed accurate prediction of outcome of more than 70% of ccRCC patients. Furthermore, the high-risk group according to this signature was shown to be a significant unfavorable prognostic factor independent of other clinical variables, such as stage and grade (Table 2B). EMT-associated miRNA/mRNA signature was successfully validated also in independent cohort of 191 RCC patients from TCGA-KIRC dataset (Fig. 4C). Finally, a large separation of predicted overall survival was observed for all stages suggesting an added value of EMT-based signature for outcome prediction (Fig. 4E).

There are some potential limitations of this study and still some issues that must be addressed in order to establish our EMT-based system as a clinical prognostic tool. The present study is a retrospective study, and the follow-up period is not yet sufficiently long to allow definitive conclusions to be made on the prognostic issues. Based on the scientific evidence in the EMT field, we decided to analyze miRNA and gene expression, but there are other classes of non-coding RNAs (lncRNAs, piRNAs, circRNAs) that may be also involved in the EMT process and reflect the postoperative prognosis in ccRCC patients. All samples used in this study were obtained in a single center, and all patients were of the same ethnicity (European descent). Therefore, these results need to be validated in a multicenter trial on independent cohorts.

In conclusion, the EMT-targeted approach enabled identification of novel miRNAs/mRNAs associated with metastasis and prognosis, and development of stage-independent prognostic model in ccRCC. We believe that this finding has clinical utility in determining which ccRCC patients will have poor prognosis lesions (all stages) and in consequent adjustment of follow-up regimens enabling detection of recurrence or metastasis early and initiate treatment in hopes of having a positive impact on the long-term survival.

## Additional Information

**How to cite this article**: Mlcochova, H. *et al*. Epithelial-mesenchymal transition-associated microRNA/mRNA signature is linked to metastasis and prognosis in clear-cell renal cell carcinoma. *Sci. Rep.* **6**, 31852; doi: 10.1038/srep31852 (2016).

## Supplementary Material

Supplementary Information

## Figures and Tables

**Table 1:** Clinical characteristics of the patients.

	Discovery phase		Training phase		Validation phase	
	microRNA	mRNAs	microRNA	mRNAs	microRNA	mRNAs
Patients included	29	13	75	47	126	135
Tumor tissue samples	29	13	75	47	126	135
Renal parenchyma	—	—	10	8	40	41
Gender						
Male	17 (58.6)	8 (61.5)	51 (68)	34 (72.3)	84 (66.7)	88 (65.2)
Female	12 (41.4)	5 (38.5)	24 (32)	13 (27.7)	42 (33.3)	47 (34.8)
Age at surgery						
Median	66	66	64	61	65	65
range	35–80	35–80	31–84	31–82	34–86	33–86
pT-stage						
T1a	7 (24.1)	4 (30.8)	15 (20)	12 (25.5)	45 (35.7)	48 (35.6)
T1b	3 (10.3)	1 (7.7)	23 (30.7)	14 (29.8)	24 (19)	28 (20.7)
T2	3 (10.3)	1 (7.7)	9 (12)	5 (10.6)	15 (11.9)	18 (13.3)
T3a	9 (31)	3 (23.1)	12 (16)	8 (17)	21 (16.7)	20 (14.8)
T3b	4 (13.8)	2 (15.4)	13 (17.3)	6 (12.8)	13 (10.3)	15 (11.1)
T4	0 (0)	0 (0)	1 (1.3)	1 (2.1)	8 (6.3)	6 (4.4)
NA	3 (10.3)	2 (15.4)	2 (2.7)	1 (2.1)	0 (0)	0 (0)
pN-stage						
N0	26 (89.7)	11 (84.6)	65 (86.7)	41 (87.2)	106 (84.1)	115 (85.2)
N1	0 (0)	0 (0)	4 (5.3)	3 (6.4)	13 (10.3)	15 (11.1)
NA	3 (10.3)	2 (15.4)	6 (8)	3 (6.4)	7 (5.6)	5 (3.7)
Metastasis						
M0	25 (86.2)	11 (84.6)	58 (77.3)	38 (80.9)	88 (69.8)	97 (71.9)
M1	1 (3.4)	0 (0)	15 (20)	8 (17)	37 (29.4)	37 (27.4)
NA	3 (10.3)	2 (15.4)	2 (2.7)	1 (2.1)	1 (0.8)	1 (0.7)
TNM Stage						
I	10 (34.5)	5 (38.5)	34 (45.3)	24 (51.1)	63 (50)	67 (49.6)
II	3 (10.3)	1 (7.7)	8 (10.7)	5 (10.6)	12 (9.5)	14 (10.4)
III	12 (41.4)	5 (38.5)	15 (20)	8 (17)	12 (9.5)	15 (11.1)
IV	1 (3.4)	0 (0)	15 (20)	8 (17)	36 (28.6)	36 (26.7)
NA	3 (10.3)	2 (15.4)	3 (4)	2 (4.3)	3 (28.6)	3 (2.2)
Fuhrman grade						
1	5 (17.2)	3 (23.1)	13 (17.3)	9 (19.1)	24 (19)	25 (18.5)
2	17 (58.6)	8 (61.5)	34 (45.3)	21 (44.7)	51 (40.5)	56 (41.5)
3	5 (17.2)	1 (7.7)	22 (29.3)	14 (29.8)	37 (29.4)	40 (29.6)
4	1 (3.4)	0 (0)	5 (6.7)	2 (4.3)	13 (10.3)	13 (9.6)
NA	1 (3.4)	1 (7.7)	1 (1.3)	1 (2.1)	1 (0.8)	1 (0.7)

N/A–not available.

Percentage is presented in brackets.

**Table 2:**
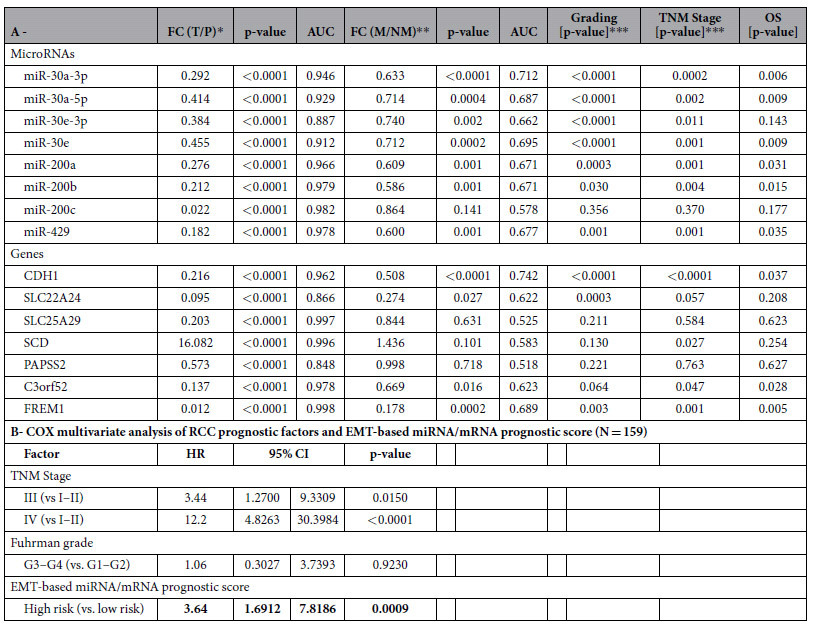
Summary of the validation of EMT-associated miRNAs/mRNAs (A) and COX multivariate analysis of RCC prognostic factors and EMT-based prognostic score (B).

^**^FC = fold change, M = metastatic primary tumors (stage III-pN+/IV), NM = non-metastatic primary tumors (stage I/II).

^***^Kruskal–Wallis test.

OS, overall survival.
