# Supplementary material for: Epithelial-mesenchymal transition-associated microRNA/mRNA signature is linked to metastasis and prognosis in clear-cell renal cell carcinoma
Source: Sci Rep. 2016 Aug 23;6:31852. doi: 10.1038/srep31852 (PMC4994011; doi:10.1038/srep31852)
Supplement: Supplementary Information [file srep31852-s1.pdf]

Epithelial-mesenchymal transition-associated microRNA/mRNA signature is linked to metastasis and prognosis in clear-cell renal cell carcinoma

Hana Mlcochova, Tana Machackova, Anja Rabien, Lenka Radova, Pavel Fabian, Robert Iliev, Katerina Slaba, Alexandr Poprach, Ergin Kilic, Michal Stanik, Martina Redova-Lojova, Marek Svoboda, Jan Dolezel, Rostislav Vyzula, Klaus Jung, Ondrej Slaby

Supplementary data

Supplementary Table S1 List of the antibodies used for immunofluorescence analysis of EMT markers.

Probe name	Primary antibody	Clone	Dilution	Cat	Lot	Company	Secondary antibody
**E-cadherin**	Purified Mouse Anti-E-Cadherin	CDH1	1:50	610182	2104735	BD Transduction Laboratories™	alpha m Alexa 488 (1:100)*
**CK-18**	Mouse Anti-Cytokeratin 18	DC-10	1:1000	MU143-UC	MU1430413	BioGenex	alpha m Alexa 488 (1:100) *
**CK-19**	Rabbit Anti-Cytokeratin 19	EP1580Y	1:500	1968-1 (ab52625)	GR26970-6	Epitomics (Abcam)	alpha rb Alexa 594 (1:50) **
**Vimentin**	Monoclonal Mouse Anti-Vimentin	V9	1:1000	M0725	20002900	Dako	alpha m Alexa 488 (1:100) *
**S100A4**	Rabbit Anti-S100A4 antibody	EPR2761(2)	1:250	ab124805	GR154927-1	Abcam	alpha rb Alexa 594 (1:50) **

*Alexa Fluor 488 F(ab')2 Fragment of Goat anti- Mouse IgG (H+L). Code A-11017. Lot 1107471. Life Technologies

**Alexa Fluor 594 F(ab')2 Fragment of Goat anti- Rabbit IgG (H+L). Code A-11072. Lot 948493. Life Technologies

Supplementary Table S2 List of miRNA expression qPCR assays used in training and validation phase of the study.

miRBase ID Release version 21	miRBase Accession No.	AB assay name	AB assay ID	Mature miRNA Sequence
hsa-miR-17-3p	MIMAT0000071	hsa-miR-17*	2421	ACUGCAGUGAAGGCACUUGUAG
hsa-miR-26a-1-3p	MIMAT0004499	hsa-miR-26a-1*	2443	CCUAUUCUUGGUUACUUGCACG
hsa-miR-30a-3p	MIMAT0000088	hsa-miR-30a-3p	416	CUUUCAGUCGGAUGUUUGCAGC
hsa-miR-30a-5p	MIMAT0000087	hsa-miR-30a-5p	417	UGUAAACAUCCUCGACUGGAAG
hsa-miR-30b-5p	MIMAT0000420	hsa-miR-30b	602	UGUAAACAUCCUACACUCAGCU
hsa-miR-30c-5p	MIMAT0000244	hsa-miR-30c	419	UGUAAACAUCCUACACUCUCAGC
hsa-miR-30d-3p	MIMAT0004551	hsa-miR-30d*	2305	CUUUCAGUCAGAUGUUUGCUGC
hsa-miR-30e-5p	MIMAT0000692	hsa-miR-30e	2223	UGUAAACAUCCUUGACUGGAAG
hsa-miR-30e-3p	MIMAT0000693	hsa-miR-30e-3p	422	CUUUCAGUCGGAUGUUUACAGC
hsa-miR-130b-5p	MIMAT0004680	hsa-miR-130b*	2114	ACUCUUUCCCUGUUGCACUAC
hsa-miR-141-3p	MIMAT0000432	hsa-miR-141	463	UAACACUGUCUGGUAAAGAUGG
hsa-miR-192-3p	MIMAT0004543	hsa-miR-192*	2272	CUGCCAAUUCCAUAGGUCACAG
hsa-miR-193b-3p	MIMAT0002819	hsa-miR-193b	2367	AACUGGCCCUCAAAGUCCCGCU
hsa-miR-200a-3p	MIMAT0000682	hsa-miR-200a	502	UAACACUGUCUGGUAACGAUGU
hsa-miR-200a-5p	MIMAT0001620	hsa-miR-200a*	1011	CAUCUUACCGGACAGUGCUGGA
hsa-miR-200b-3p	MIMAT0000318	hsa-miR-200b	2251	UAAUACUGCCUGGUAAUGAUGA
hsa-miR-200b-5p	MIMAT0004571	hsa-miR-200b*	2274	CAUCUUACUGGGCAGCAUUGGA
hsa-miR-200c-3p	MIMAT0000617	hsa-miR-200c	2300	UAAUACUGCCGGGUAAUGAUGG A
hsa-miR-215-5p	MIMAT0000272	hsa-miR-215	518	AUGACCUAUGAAUUGACAGAC
hsa-miR-429	MIMAT0001536	hsa-miR-429	1024	UAAUACUGUCUGGUAAAACCGU
hsa-miR-571	MIMAT0003236	hsa-miR-571	1613	UGAGUUGGCCAUCUGAGUGAG
hsa-miR-630	MIMAT0003299	hsa-miR-630	1563	AGUAUUCUGUACCAGGGAAGGU
hsa-miR-632	MIMAT0003302	hsa-miR-632	1572	GUGUCUGCUUCCUGUGGGA
hsa-miR-770-5p	MIMAT0003948	hsa-miR-770-5p	2002	UCCAGUACCACGUGUCAGGGCCA
RNU48	NCBI Accession Number: NR_002745	RNU48	1006	GATGACCCCAGGTAACTCTGAGTG TGTCGCTGATGCCATCACCGCAGC GCTCTGACC

Supplementary Table S3 List of gene expression qPCR assays used in training and validation phase of the study.

Gene symbol	Entrez Gene ID	Gene name	Assay name	Assay ID
SCD	6319	stearoyl-CoA desaturase (delta-9-desaturase)	SCD	Hs01682761_m1
PAPSS2	9060	3'-phosphoadenosine 5'-phosphosulfate synthase 2	PAPSS2	Hs00989928_m1
SEPT14	346288	septin 14	SEPT14	Hs01121472_m1
KMO	8564	kynurenine 3-monooxygenase (kynurenine 3-hydroxylase)	KMO	Hs00175738_m1
ZNF343	79175	zinc finger protein 343	ZNF343	Hs00375483_m1
CLVS2	134829	clavesin 2	CLVS2	Hs01383800_m1
CALCR	799	calcitonin receptor	CALCR	Hs01016882_m1
PCDHB10	56126	protocadherin beta 10	PCDHB10	Hs01585827_s1
C3orf52	79669	chromosome 3 open reading frame 52	C3orf52	Hs00226345_m1
FREM1	158326	FRAS1 related extracellular matrix 1	FREM1	Hs00381549_m1
SLC7A5	8140	solute carrier family 7 (amino acid transporter light chain. L system), member 5	SLC7A5	Hs01001183_m1
SLC22A24	283238	solute carrier family 22, member 24	SLC22A24	Hs00543210_m1
SLC17A3	10786	solute carrier family 17 (organic anion transporter), member 3	SLC17A3	Hs00198361_m1
SLC25A29	123096	solute carrier family 25 (mitochondrial carnitine/acylcarnitine carrier), member 29	SLC25A29	Hs00936827_m1
ITGB1BP1	9270	integrin beta 1 binding protein 1	ITGB1BP1	Hs01592320_g1
CDH1	999	cadherin 1, type 1, E-cadherin (epithelial)	CDH1	Hs01023894_m1
CDH12	1010	cadherin 12, type 2 (N-cadherin 2)	CDH12	Hs00362037_m1
VIM	7431	vimentin	VIM	Hs00958111_m1
ZEB1	6935	zinc finger E-box binding homeobox 1	ZEB1	Hs00232783_m1
ZEB2	9839	zinc finger E-box binding homeobox 2	ZEB2	Hs00207691_m1
S100A4	6275	S100 calcium binding protein A4	S100A4	Hs00243202_m1
PPIA	5478	peptidylprolyl isomerase A (cyclophilin A)	PPIA	Hs99999904_m1

Supplementary Table S4 Results of immunofluorescence staining of EMT markers in ccRCC samples.

	Marker	Weak positivity (%)	Strong positivity (%)
**Epithelial markers**	E-cadherin (CDH1)	74	26
	CK-18	54.8	45.2
	CK-19	37	63
**Mesenchymal markers**	vimentin	30.1	69.9
	S100A4	15.1	84.9

Supplementary Table S5 List of miRNAs with significantly different expression between EMT+ and EMT- groups of ccRCC patients (P < 0.05).

miRNA	logFC	P-Value
**miR-200b***	**-2.921**	**0.003**
**miR-30b**	**-1.019**	**0.005**
**miR-200a**	**-1.840**	**0.006**
**miR-200b**	**-1.397**	**0.010**
**miR-30a***	**-1.195**	**0.014**
**miR-30e***	**-1.042**	**0.019**
**miR-30e-3p**	**-1.019**	**0.019**
miR-192	-1.530	0.019
**miR-429**	**-1.324**	**0.020**
**miR-130b***	**1.730**	**0.021**
**miR-30c**	**-0.836**	**0.022**
miR-190	-1.360	0.024
miR-486-3p	-2.110	0.025
miR-564	1.777	0.026
**miR-630**	**1.588**	**0.027**
**miR-17***	**-1.657**	**0.030**
miR-204	-1.723	0.031
**miR-30a**	**-1.044**	**0.031**
miR-139-5p	-1.395	0.031
**miR-192***	**-1.308**	**0.034**
miR-194	-1.332	0.036
**miR-200a***	**-1.700**	**0.042**
miR-140-3p	-0.859	0.042
miR-505	-2.056	0.043
miR-135a	-2.377	0.046
miR-486-5p	-1.334	0.047

MiRNAs used in training phase are bold.

Supplementary Table S6 List of genes with significantly different expression between EMT+ and EMT- groups of ccRCC patients ($P < 0.005$).

Gene symbol	Gene name	logFC	AveExpr	P.Value
**SCD**	**stearoyl-CoA desaturase (delta-9-desaturase)**	**1,04**	**7,33**	**0,0001**
**PAPSS2**	**3'-phosphoadenosine 5'-phosphosulfate synthase 2**	**1,21**	**6,14**	**0,0003**
**SEPT14**	**septin 14**	**0,91**	**5,83**	**0,0004**
**KMO**	**kynurenine 3-monooxygenase (kynurenine 3-hydroxylase)**	**2,26**	**5,58**	**0,0004**
NCRNA00266	non-protein coding RNA 266	0,84	6,60	0,0005
**ZNF343**	**zinc finger protein 343**	**0,50**	**5,05**	**0,0007**
**CLVS2**	**clavesin 2**	**-0,56**	**3,32**	**0,0010**
**CALCR**	**calcitonin receptor**	**1,53**	**3,91**	**0,0011**
**PCDHB10**	**protocadherin beta 10**	**0,68**	**5,08**	**0,0013**
SEPT14	septin 14	0,79	6,62	0,0013
**C3orf52**	**chromosome 3 open reading frame 52**	**-0,86**	**4,47**	**0,0014**
**FREM1**	**FRAS1 related extracellular matrix 1**	**-1,01**	**4,35**	**0,0015**
PCMTD2	protein-L-isoaspartate (D-aspartate) O-methyltransferase domain containing 2	0,76	5,36	0,0015
PCMTD2	protein-L-isoaspartate (D-aspartate) O-methyltransferase domain containing 2	0,76	5,36	0,0015
ERV3	endogenous retroviral sequence 3	0,61	7,06	0,0016
GRB7	growth factor receptor-bound protein 7	-0,53	5,09	0,0018
PCDHB13	protocadherin beta 13	0,79	4,02	0,0019
TTC1	tetratricopeptide repeat domain 1	0,69	5,45	0,0019
PCDHB14	protocadherin beta 14	0,69	5,48	0,0019
RNF145	ring finger protein 145	0,64	6,14	0,0020
PCDHB8	protocadherin beta 8	0,78	3,11	0,0021
TMEM167A	transmembrane protein 167A	0,54	6,61	0,0021
CAPG	capping protein (actin filament)	0,75	6,20	0,0022
GRK1	G protein-coupled receptor kinase 1	-0,42	4,11	0,0022
C17orf108	chromosome 17 open reading frame 108	0,54	2,88	0,0023
TXNDC9	thioredoxin domain containing 9	0,46	4,85	0,0023
PNPT1	polyribonucleotide nucleotidyltransferase 1	0,52	5,63	0,0024
ZNF117	zinc finger protein 117	0,65	6,01	0,0024
AIF1L	allograft inflammatory factor 1-like	-0,92	5,52	0,0024
SLITRK5	SLIT and NTRK-like family, member 5	-0,55	4,65	0,0027
FARSB	phenylalanyl-tRNA synthetase, beta subunit	0,55	5,85	0,0028
CCL28	chemokine (C-C motif) ligand 28	1,45	6,51	0,0028
VKORC1L1	vitamin K epoxide reductase complex, subunit 1-like 1	0,64	5,07	0,0030
BET1	blocked early in transport 1 homolog (S. cerevisiae)	0,50	5,34	0,0031
**SLC7A5**	**solute carrier family 7 (cationic amino acid transporter, y+ system), member 5**	**0,80**	**5,46**	**0,0031**
ZFP36L1	zinc finger protein 36, C3H type-like 1	-0,60	8,83	0,0031
PDCL3	phosducin-like 3	0,54	4,63	0,0032
FLJ32742	hypothetical locus FLJ32742	-1,08	3,57	0,0033
FRG2C	FSHD region gene 2 family, member C	-0,44	3,16	0,0034
ANKRD34B	ankyrin repeat domain 34B	-0,39	2,62	0,0035
BCL2	B-cell CLL/lymphoma 2	-0,62	6,67	0,0035
OR10W1	olfactory receptor, family 10, subfamily W, member 1	-0,37	3,94	0,0036

HFE	hemochromatosis	0,53	4,75	0,0038
ERGIC1	endoplasmic reticulum-golgi intermediate compartment (ERGIC) 1	0,53	7,70	0,0038
**SLC22A24**	**solute carrier family 22, member 24**	**-0,81**	**3,66**	**0,0038**
PCDHB18	protocadherin beta 18 pseudogene	0,65	5,24	0,0038
TMCO2	transmembrane and coiled-coil domains 2	-0,37	3,06	0,0038
NAA11	N(alpha)-acetyltransferase 11, NatA catalytic subunit	-0,40	4,15	0,0039
RASL10A	RAS-like, family 10, member A	-0,45	4,47	0,0039
CYP21A2	cytochrome P450, family 21, subfamily A, polypeptide 2	-0,46	4,90	0,0040
ODZ2	odz, odd Oz/ten-m homolog 2 (Drosophila)	-0,59	4,04	0,0041
AGTPBP1	ATP/GTP binding protein 1	0,49	4,98	0,0042
KIAA1715	KIAA1715	0,89	6,59	0,0043
OSR2	odd-skipped related 2 (Drosophila)	-0,43	4,28	0,0044
**SLC17A3**	**solute carrier family 17 (sodium phosphate), member 3**	**-2,47**	**7,41**	**0,0044**
AJAP1	adherens junctions associated protein 1	-0,60	4,61	0,0044
PDE8B	phosphodiesterase 8B	-0,57	4,77	0,0044
AMIGO1	adhesion molecule with Ig-like domain 1	-0,46	5,67	0,0045
ANPEP	alanyl (membrane) aminopeptidase	-1,32	8,63	0,0045
TM6SF2	transmembrane 6 superfamily member 2	-0,71	5,80	0,0046
PTCH2	patched 2	0,81	2,91	0,0046
FUT3	fucosyltransferase 3 (galactoside 3(4)-L-fucosyltransferase, Lewis blood group)	-0,56	4,08	0,0046
RASA3	RAS p21 protein activator 3	0,51	5,62	0,0047
ANKRD2	ankyrin repeat domain 2	-0,37	4,50	0,0050

Supplementary Table S7 Evaluation of the EMT-associated miRNAs the training phase of the study.

	FC (tu/par)	p-value	Grading [p-value]	Stage [p-value]	OS [p-value]
miR-17*	1.152	0.053	0.031	0.034	0.400
**miR-30a-3p**	**0.328**	**< 0.0001**	**0.021**	**0.065**	**0.016**
**miR-30a-5p**	**0.408**	**0.0003**	**0.045**	**0.125**	**0.050**
miR-30b	0.550	0.002	0.016	0.541	0.331
miR-30c	0.408	< 0.0001	0.028	0.409	0.036
miR-30d*	0.307	< 0.0001	0.017	0.472	0.177
**miR-30e**	**0.495**	**0.001**	**0.037**	**0.072**	**0.032**
**miR-30e-3p**	**0.436**	**0.0002**	**0.046**	**0.104**	**0.304**
miR-130b	1.333	0.006	0.303	0.633	0.188
miR-141	0.014	< 0.0001	0.842	0.795	0.957
miR-192*	0.641	0.455	0.998	0.981	0.313
miR-193b	0.906	0.948	0.033	0.188	0.092
**miR-200a**	**0.354**	**< 0.0001**	**0.218**	**0.050**	**0.002**
miR-200a*	0.357	< 0.0001	0.287	0.088	0.086
**miR-200b**	**0.244**	**< 0.0001**	**0.368**	**0.018**	**0.019**
miR-200b*	0.316	< 0.0001	0.519	0.138	0.014
**miR-200c**	**0.028**	**< 0.0001**	**0.671**	**0.357**	**0.224**
miR-215	0.746	0.806	0.880	0.466	0.550
**miR-429**	**0.161**	**< 0.0001**	**0.050**	**0.009**	**0.005**
miR-630	ND	ND	ND	ND	ND

ND – not detectable

MiRNAs used in validation phase are bold.

Supplementary Table S8 Evaluation of the EMT-associated genes in the training phase of the study.

	FC (tu/par)	p-value	Grading [p-value]	Stage [p-value]	OS [p-value]
**CDH1**	**0.291**	**< 0.0001**	**0.047**	**0.209**	**0.004**
CHD12	0.014	< 0.0001	ND	0.715	0.584
VIM	2.609	< 0.0001	0.198	0.584	0.700
ZEB1	1.369	0.379	0.038	0.232	0.156
ZEB2	0.959	0.914	0.008	0.155	0.157
S100A4	0.934	0.678	0.304	0.296	0.079
SLC7A5	1.194	0.489	0.239	0.675	0.144
**SLC22A24**	**0.159**	**0.002**	**0.011**	**0.224**	**0.155**
**SLC25A29**	**0.228**	**< 0.0001**	**0.572**	**0.455**	**0.042**
SLC17A3	2.514	0.224	0.131	0.732	0.111
**SCD**	**21.447**	**< 0.0001**	**0.278**	**0.014**	**0.093**
**PAPSS2**	**0.533**	**0.0002**	**0.259**	**0.144**	**0.000**
SEPT14	ND	ND	ND	ND	ND
KMO	1.713	0.431	0.059	0.071	0.438
CLVS2	3.664	0.256	ND	0.219	0.846
ZNF343	0.982	0.644	0.260	0.644	0.178
CALCR	0.693	0.445	0.401	0.618	0.523
PCDHB10	0.958	0.445	0.038	0.226	0.019
**C3orf52**	**0.148**	**< 0.0001**	**0.153**	**0.001**	**0.020**
**FREM1**	**0.026**	**< 0.0001**	**0.007**	**0.089**	**0.033**
ITGB1BP1	0.635	< 0.0001	0.461	0.363	0.175

ND – not detectable

Genes used in phase 3 are in bold

Supplementary Table S9 Significant survival predictors (EMT-associated miRNAs/mRNAs) selected by Cox regression model by stepwise selection.

fold	Significant predictors selected for Cox regression models by bidirectional stepwise selection	AUC for training set (9/10 dataset)	AUC for validation set (1/10 dataset)
1	miR-200a, miR-200b, miR-200c, miR-429, miR-30a-3p, CDH1, PAPSS2	0.72	0.68
2	miR-200a, miR-200b, miR-200c, miR-429, miR-30a-3p, miR-30e-3p, CDH1	0.74	0.70
3	miR-200a, miR-200b, miR-200c, miR-429, miR-30a-3p, CDH1, SLC7A5, PAPSS2	0.71	0.66
4	miR-200a, miR-200b, miR-200c, miR-429, CDH1, PAPSS2	0.70	0.76
5	miR-200a, miR-200b, miR-200c, miR-429, miR-30a-5p, miR-30a-3p, CDH1, SLC25A29, PAPSS2, C3orf52	0.76	0.80
6	miR-200a, miR-200b, miR-200c, miR-429, miR-30a-3p, CDH1, SLC7A5, SCD, PAPSS2, C3orf52	0.76	0.80
7	miR-200a, miR-200b, miR-200c, miR-429, miR-30a-5p, miR-30a-5p, miR-30a-3p, CDH1, SLC7A5, SCD, PAPSS2, C3orf52	0.75	0.66
8	miR-200a, miR-200b, miR-200c, miR-429, CDH1, PAPSS2, C3orf52	0.70	0.81
9	miR-200a, miR-200b, miR-200c, miR-429, miR-30a,3p, miR-30e-3p, CDH1, PAPSS2, C3orf52	0.74	0.70
10	miR-200a, miR-200b, miR-200c, miR-429, miR-30a-3p, miR-30e-3p, CDH1, SLC7A5, PAPSS2	0.73	0.63

Supplementary Code 1

Pseudocode of predictive analyses: Cox model & 5year overall survival

1. Dataset = patients from both cohorts with expression levels of 18 candidate transcripts (8 miRNAs and 10 genes)
2. denote: 0 = patient died until 5years, 1 = patient censored until 5years, 2 = patient survived 5 years timepoint
3. split patients into 10 folds with equivalent proportions of died until 5years (0), censored during 5years (1) and alive in 5years timepoint.
4. repeat for i in 1:10
   a) VALIDATION_i = patients in fold i
   b) TRAINING_i = patients in all remaining folds (except i-th fold)
   c) MODEL_i = results of stepwise selection for Cox model applied on dataset TRAINING_i, with 18 candidate predictors (model with minimal Akaike information criteria)
   d) ROC_i = survivalROC analysis for MODEL_i on dataset VALIDATION_i in timepoint t=5years
   e) SIGNCANDIDATE_i = candidates from MODEL_i
5. CANDIDATE_FINAL = candidate transripts, which occur at least 5-times in SIGNCANDIDATE _1, …, SIGNCANDIDATE _10
6. FULL_MODEL = Cox model on all patients with predictors CANDIDATE_FINAL
7. survivalROC analysis on FULL_MODEL in timepoint t=5years; output: THRESHOLD=1.3497 based on Youden index and black ROC curve in fig. 4a,
8. bootstrap for censored data applied on FULL_MODEL with 2000 replicate; output in table: standard errors of AUC, sensitivity, specificity and accuracy for FULL_MODEL
9. AUC, sensitivity, specificity and accuracy for ROC_1, … ,ROC_10 in timepoint t=5years; output in table: standard errors of AUC, sensitivity, specificity and accuracy
10. PRED_INDV = individual predictions of FULL_MODEL
11. CATEGORY – split patients into 2 subsets
   a) "HIGHrisk" – patients with PRED_INDV >= THRESHOLD
   b) "LOWrisk" – patients with PRED_INDV < THRESHOLD
12. plot Kaplan-Meier curves for CATEGORY in fig.4b
13. CLIN_MODEL=Cox model with predictors gender and stage

14 plot individual predictions of CLIN_MODEL with fixed gender::MALE; output fig. 4d

15. MIXED_MODEL = Cox model with predictors gender, stage, CATEGORY
16. plot individual predictions of MIXED_MODEL with fixed gender::MALE and stage::1 (red lines), stage::2 (blue lines), stage::3 (green lines), stage::4 (black lines); output fig. 4e

Supplementary figures

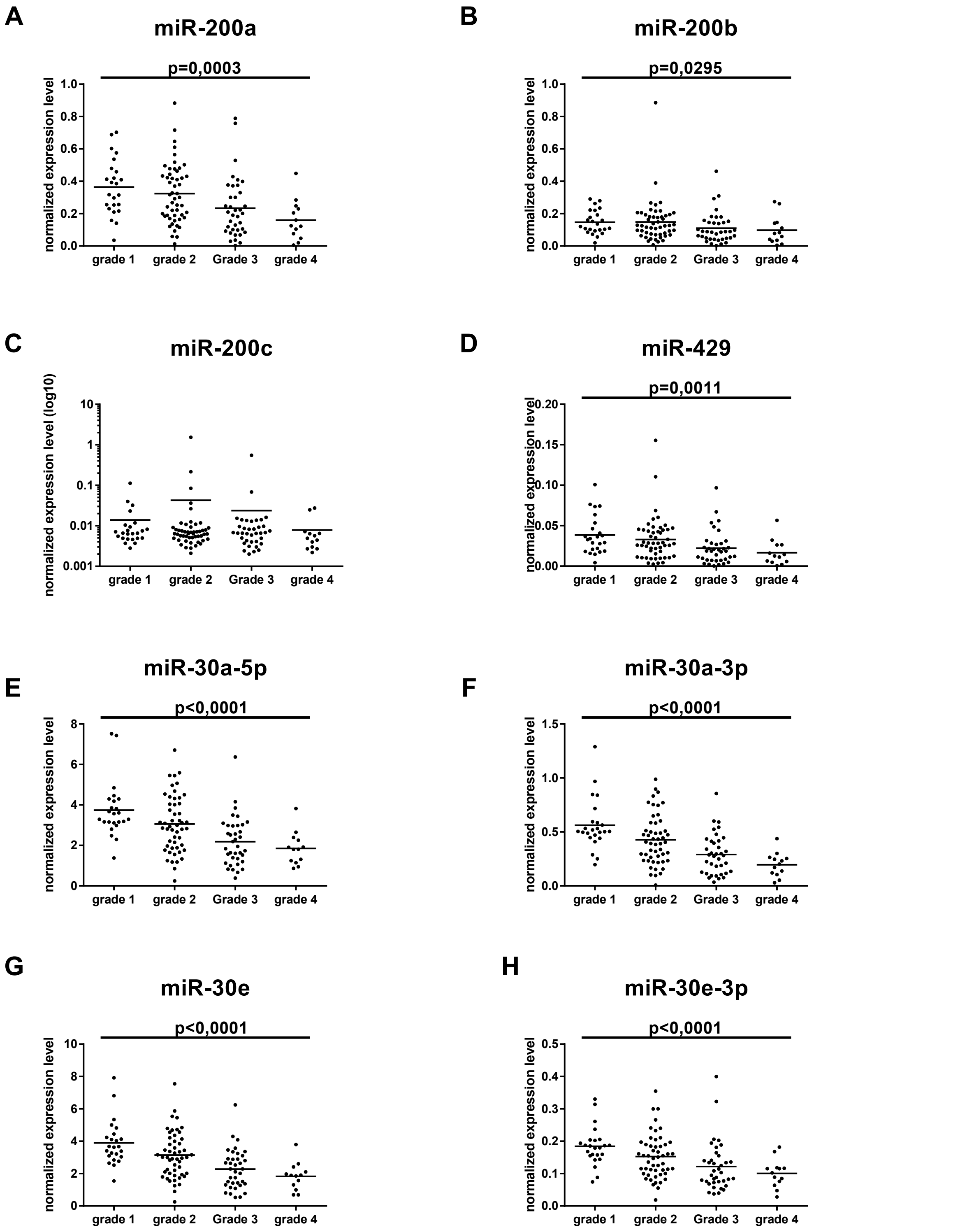

Supplementary Figure S1 EMT-associated miRNAs in relationship to Fuhrman grades in the validation phase of the study. P-value was reached by Kruskal-Wallis test.

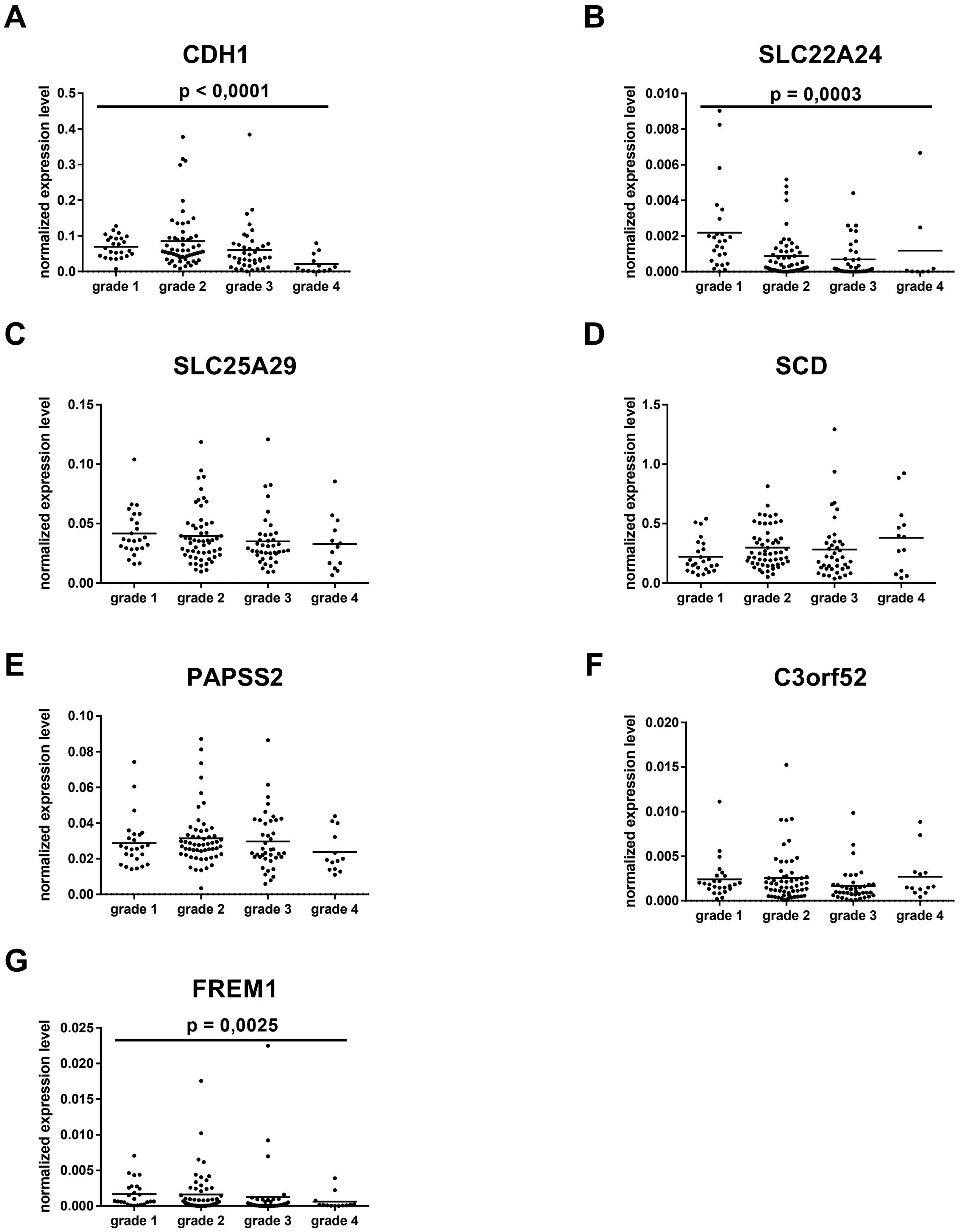

Supplementary Figure S2 EMT-associated genes in relationship to Fuhrman grades in the validation phase of the study. P-value was reached by Kruskal-Wallis test.
